# Trends in oesophageal cancer mortality in Montenegro, 1990–2018

**DOI:** 10.1093/eurpub/ckae080

**Published:** 2024-05-22

**Authors:** Mirjana Nedović Vuković, Marina Jakšić, Brigita Smolović, Miloš Lukić, Zoran Bukumirić

**Affiliations:** Department of Health Statistics, Center for Health System Evidence and Research in Public Health, Institute for Public Health of Montenegro, Podgorica, Montenegro; Faculty of Medicine, University of Montenegro, High School for Nurses in Berane, Podgorica, Montenegro; Faculty of Medicine, University of Montenegro, High School for Nurses in Berane, Podgorica, Montenegro; Department of Pathophysiology and Laboratory Medicine, Institute for Children’s Diseases, Clinical Center of Montenegro, Podgorica, Montenegro; Faculty of Medicine, University of Montenegro, High School for Nurses in Berane, Podgorica, Montenegro; Department of Internal Medicine, Department of Gastroenterology and Hepatology, Internal Clinic, Clinical Center of Montenegro, Podgorica, Montenegro; Department of Internal Medicine, Department of Gastroenterology and Hepatology, Internal Clinic, Clinical Center of Montenegro, Podgorica, Montenegro; Faculty of Medicine, Institute of Medical Statistics and Informatics, University of Belgrade, Belgrade, Serbia

## Abstract

**Background:**

Oesophageal cancer (OC) is a significant public health issue, despite the decreasing trends in OC mortality rates observed globally in the past decades. The objective of our study is to analyze the pattern of OC mortality in Montenegro between 1990 and 2018 and contribute to the development of a national long-term strategy for the prevention and control of this malignancy.

**Methods:**

The data on OC death cases in Montenegro between 1990 and 2018 were collected. The mortality rates were standardized according to the World Standard Population. The Joinpoint, Linear and Poisson regressions were applied to analyze the OC mortality trend.

**Results:**

Joinpoint regression analysis showed an increase in death rates for men and the overall level which were not statistically significant. However, the number of cases increases significantly with an average annual percentage change (AAPC) increase of 2.6% for the overall level [AAPC (95% CI)=2.6 (1.0–4.2); *P* = 0.002] at the expense of the increase in men, which on average was 2.6% annually [AAPC (95%CI) = 2.6 (1.2–4.1); *P* = 0.001]. The age groups 55–64 and 65–74 have the highest percentage of deaths cases from OC with 30.6% and 31.4%, respectively.

**Conclusion:**

Montenegro has witnessed a recent increase in the number of deaths from OC, although the mortality rates remain stable. National strategies to further reduce mortality rates for OC are necessary. Individuals aged 55–64 and 65–74 need specific attention during the ongoing monitoring of this cancer.

## Introduction

Oesophageal cancer (OC) is the sixth leading cause of death worldwide, with 544 076 deaths recorded in 2020, out of which 374 313 were men and 169 763 were women. It accounts for 1 in every 18 cancer-related deaths. Around 70% of the cases occur in men, and there is a 2–3 times higher mortality rate in men than women. The mortality rates are higher in developed countries for men, but comparable for women.^1–3^

In 2019, mortality rates for men were 9.68, while for women were 3.02.^4^^,^^5^ OC has shown a downward trend in mortality globally from 1990 to 2019, although the burden of the disease varies greatly by region and country.^3–5^ It is the most geographically differentiated tumour.^4^ The two main histological subtypes of OC are adenocarcinoma (EAC) and squamous cell carcinoma (SCC), and the risk factors vary depending on the type.^6^

OC is a complex and multifactorial disease, with different types of cancer having distinct risk factors.^5^^,^^7^^,^^8^ For instance, SCC is more prevalent in males, individuals with a family history of the disease, smokers, frequent alcohol consumers and those with specific dietary habits.^7^ Additionally, low socioeconomic status, exposure to nitrosamines and the consumption of very hot beverages can heighten the risk of developing oesophageal SCC.^8^ Moreover, deficiencies in vitamin C, vitamin E and folate can also increase the risk of SCC.^9^ However, smoking and alcohol consumption remain the two most significant risk factors, accounting for over 70% of all SCC cases in high-income regions.^10^ For instance, in China, drinking hot tea, excessive alcohol consumption and smoking are the principal causes of high OC morbidity.^11^ On the contrary, EAC is more closely linked with gastro-oesophageal reflux disease (GERD), obesity and high blood cholesterol, while smoking and alcohol consumption are more associated with SCC among men.^5^ It has been observed that African countries, especially West Sub-Saharan Africa, have a higher burden of OC due to their diet being low in iron, magnesium, zinc and selenium.^12^^,^^13^ Obesity and GERD are two major risk factors for EAC, along with Barrett's oesophagus (BE), which is a well-known premalignant condition.^8^^,^^14–17^ Patients with BE have a risk of developing EAC that is 30–40 times greater than in the general population. Almost 86% of patients with EAC have a history of BE.^18^

Fortunately, advancements in diagnostic technology and treatment have likely contributed to reduced mortality rates in many countries.^6^

Montenegro follows the latest recommendations for GERD treatment.^19^^,^^20^ European Society of Gastrointestinal Endoscopy (ESGE) guidelines from 2023 are used for dysplasia diagnosis and follow-up in BE.^19^ According to ESGE guidelines as of 2023, BE or EAC screening is not provided in Montenegro, due to the low level of risk in the general population.^19^

Experts have suggested that screening will play a crucial role in preventing OC and reducing mortality in the future.^9^^,^^21^^,^^22^ Currently, there are no established guidelines for screening for SCC of the oesophagus.^9^^,^^22^ However, guidelines for EAC have been established, but they still lack concrete evidence in the form of randomized controlled trials comparing the implementation of screening and non-implementation of screening.^9^^,^^23^

The study aims to analyze the mortality trends associated with OC in Montenegro from 1990 to 2018 using national mortality data and regression techniques. Additionally, the research endeavors to investigate the measures being taken in Montenegro to prevent fatalities arising from OC.

## Methods

The data on OC death cases in Montenegro between 1990 and 2018 were collected using the International Classification of Diseases code 150 from the 9th edition and the code C15 from the 10th edition. Death certificates were the primary data source. The State Statistical Office provided data until 2009, which were unpublished until 1999 and published in the statistical yearbooks of the Institute for Public Health of Montenegro from 1999 to 2009. After 2009, the Institute for Public Health became the data source for causes of death.^24^ Population data were obtained from the Statistical Office of Montenegro.^25^

The mortality rates were standardized according to the World Standard Population to estimate the overall and gender-specific trends of OC. The Joinpoint regression model was used to analyze the OC mortality rate and to identify significant changes in the linear time trend. The Estimated Annual Percentage Change (EAPC) and the Average Annual Percentage Change (AAPC) were used to describe the trend changes. The Joinpoint Software, version 5.0.2-May, 2023, from the Surveillance Research Program of the United States National Cancer Institute, was used for the analysis.^26^ The regression line was adjusted to the natural logarithm of rates, using the calendar year as an independent variable, to determine the EAPC. The Grid-search method was chosen for the analysis, and the minimum number of observations for points from the end of the series to the first joinpoint was established as 3 and between two joinpoints as 4. The number of joinpoints was set between 0 and 5, and the permutation test facilitated the selection of the most fitting joinpoint model with an overall significance level of 0.05. Additionally, Linear and Poisson regressions were applied, and they were both executed in the Statistical Software for Social Sciences SPSS 26 (IBM Corp., Armonk, New York, USA). Ethical approval and consent were not required as this study was based on publicly available data.

## Results

**Figure 1:**
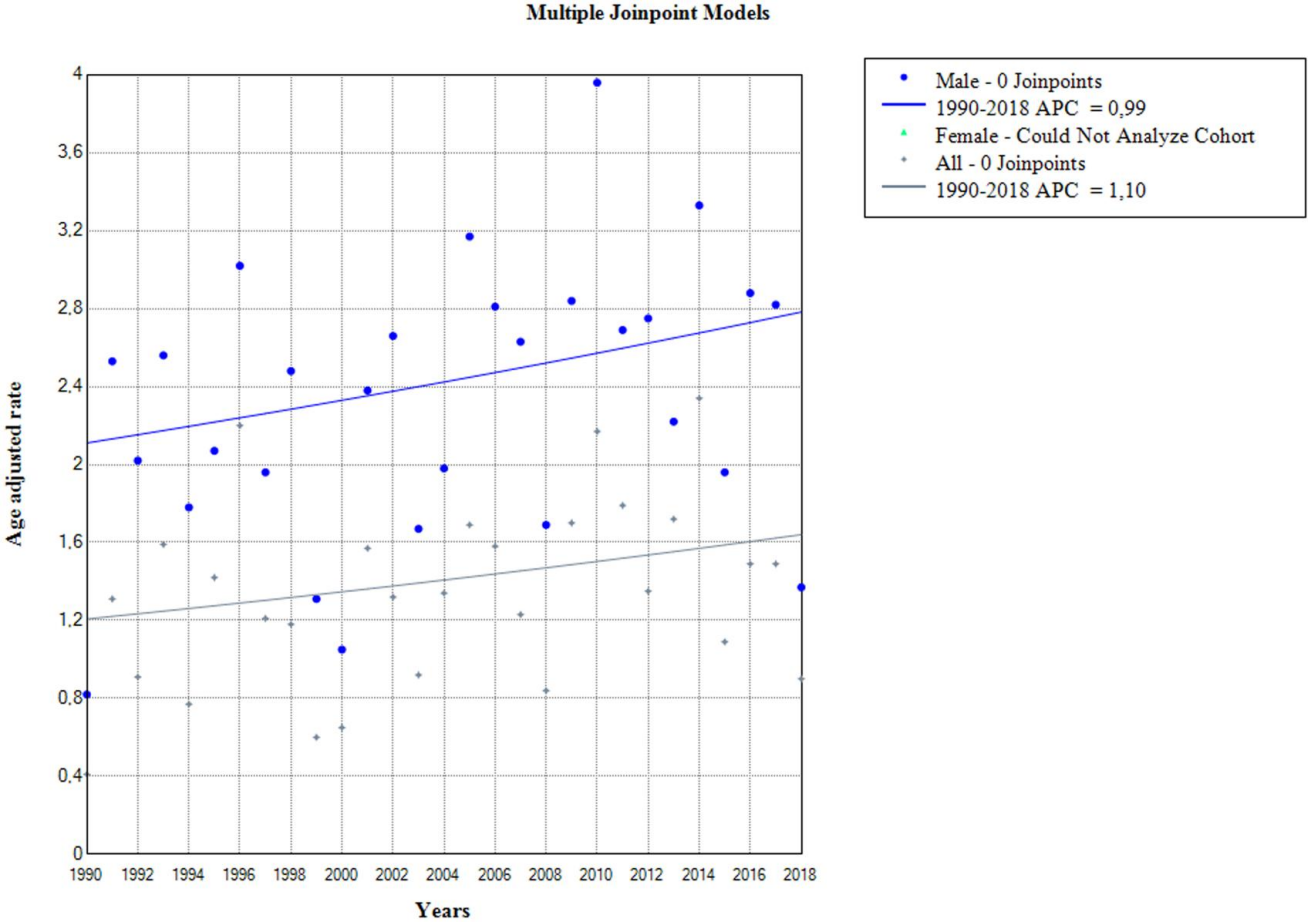
Joinpoint regression analysis of oesophageal cancer mortality (rates) in Montenegro from 1990 to 2018. AAR, age adjusted mortality rate; APC, annual percentage change.

Between 1990 and 2018, there were 353 cases of death caused by OC. The average number of deaths per year was 12 for the overall level, 9.6 for men, and 2.6 for women (table 1). The average age-standardized rate was 1.34 deaths per 100 000 population with a significantly higher rate in men (2.32/100 000 in men, 0.51 deaths/100 000 in women) (table 1). Joinpoint regression analysis showed an increase in death rates for men and the overall level (table 1, figure 1), as well as for the age group 65–74 for the same categories which were not statistically significant. For other categories (women and other age groups), joinpoint regression could not be performed due to small values for rates (table 1). Mortality rates for men and overall remained stable throughout the observation period (figure 1).

**Table 1:** Descriptive statistics for OC death cases and mortality rate in Montenegro and results of regression analyses for period 1990–2018

C15	Joinpoint regression for death cases	Joinpoint regression for mortality rate	Linear regression for mortality rate	Poisson regression for death cases	Death cases	Mortality rate	Overall death cases
			
	AAPC (95% CI)		*β* (95% CI)		Mean ± SD		
Men	2.6^a^ (1.2–4.1)	0.9 (−0.2–2.5)	0.027 (−0.003–0.058)	0.026^a^ (0.012–0.041)	9.60 ± 3.60	2.32 ± 0.71	277
25–34							2
35–44							6
45–54			0.743 (−1.043–2.530)	0.030 (−0.013–0.073)	1.69 ± 1.63	42.62 ± 39.05	49
55–64	2.2^a^ (0.4–4.1)		1.468 (−0.430–3.368)	0.028^a^ (0.003–0.053)	3.10 ± 1.47	96.86 ± 42.82	90
65–74	3.1^a^ (1.2–5.0)	1.6 (−0.9–4.1)	2.291 (−0.462–5.046)	0.031^a^ (0.005–0.057)	2.97 ± 1.43	143.48 ± 62.52	86
75–84			−0.295 (−5.872–5.282)	0.034 (−0.005–0.074)	1.24 ± 1.18	137.97 ± 120.29	36
85+							8
Women	1.9 (−1.2–5.0)		0.008 (−0.011–0.028)	0.033^a^ (0.005–0.060)	2.60 ± 2.10	0.51 ± 0.44	76
25–34							0
35–44							3
45–54			0.192 (−0.621–1.007)	0.025 (−0.040–0.091)	0.45 ± 0.74	10.93 ± 17.64	13
55–64			0.143 (−1.036–1.324)	0.017 (−0.038–0.073)	0.62 ± 0.90	17.24 ± 25.48	18
65–74			0.636 (−1.074–2.348)	0.026 (−0.021–0.074)	0.86 ± 0.99	32.38 ± 37.30	25
75–84							14
85+							3
Total	2.6^a^ (1.0–4.2)	1.1 (−0.5–3.2)	0.019 (−0.001–0.039)	0.028^a^ (0.015–0.040)	12.20 ± 4.9	1.34 ± 0.48	353
25–34							2
35–44							9
45–54	3.0 (−0.6–6.7)		0.473 (−0.582–1.530)	0.029 (−0.010–0.070)	2.14 ± 1.96	26.62 ± 23.13	62
55–64	2.1 (−0.1–4.3)		0.839 (−0.365–2.045)	0.026^a^ (0.003–0.049)	3.72 ± 1.96	55.17 ± 26.97	108
65–74	2.8^a^ (1.0–4.6)	1.9 (−0.3–4.3)	1.462 (−0.059–2.865)	0.030^a^ (0.007–0.052)	3.83 ± 1.73	81.17 ± 32.71	111
75–84	5.0^a^ (2.3–7.7)		1.442 (−0.873–3.758)	0.050^a^ (0.015–0.085)	1.72 ± 1.46	72.72 ± 51.43	50
85+							11

AAPC, Average annual percentage change; *β*, regression coefficient; CI, confidence interval.

a indicated that AAPC and *β* are statistically significantly different from zero at a *P* values less than 0.05.

**Figure 2:**
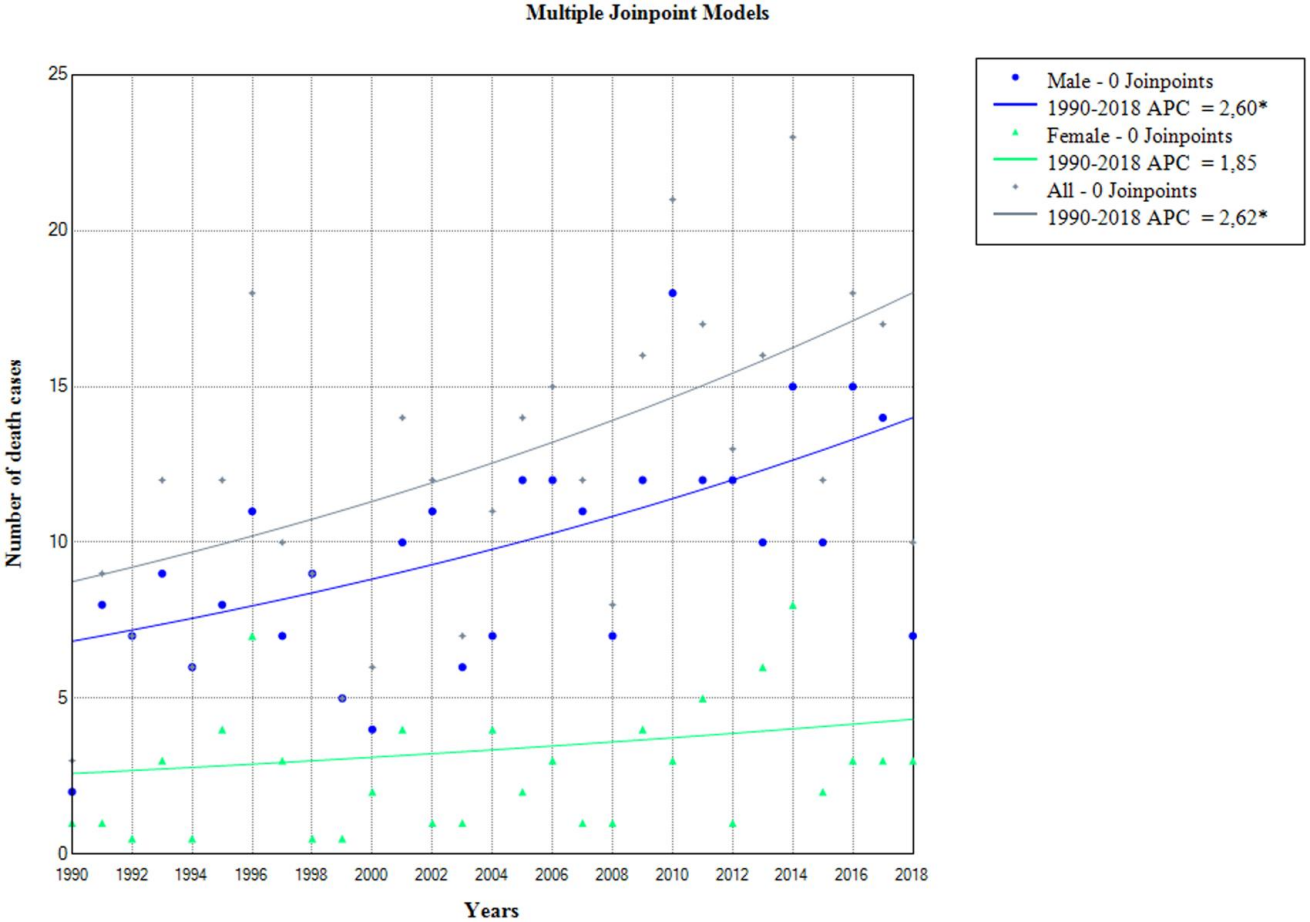
Joinpoint regression analysis of oesophageal cancer mortality (death cases) in Montenegro from 1990 to 2018. APC, annual percentage change; indicated that APC is significant different from zero at *P* values <0.05.

However, a joinpoint regression for the number of cases reveals that the number of cases increases significantly with an average annual percentage increase of 2.6% for the overall level (AAPC (95% CI) =2.6 (1.0–4.2); *P* = 0.002) at the expense of the increase in men, which on average was 2.6% annually (AAPC (95% CI) =2.6 (1.2–4.1); *P* = 0.001) (table 1, figure 2).

To assess the significance of changes when dealing with a small number of cases, Poisson regression is a more sensitive method. The results obtained from this method indicate that there was a significant increase in the number of cases during the observed period, with statistical parameter for the overall level (*β* (95% CI) =0.028 (0.015–0.040); *P* < 0.001), and for male (β (95% CI) = 0.026 (0.012–0.041); *P* < 0.001) and female (*β* (95% CI) =0.033 (0.005–0.060); *P* = 0.019) (table 1).

**Figure 3:**
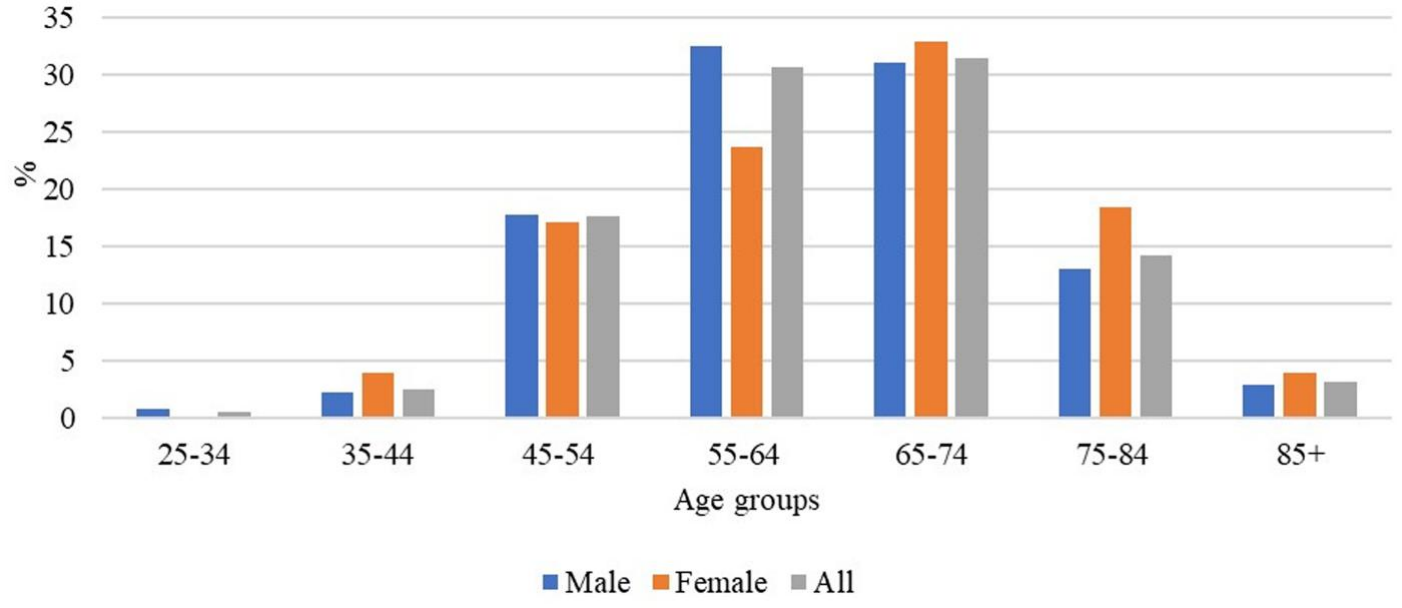
Distribution of oesophageal cancer mortality by age groups in Montenegro,1990–2018.

The age groups 55–64 and 65–74 have the highest percentage of cases and deaths from OC, with 30.6% and 31.4%, respectively (figure 3). Joinpoint regression analysis has found a statistically significant increase in the number of death cases in males in these age groups, with an average annual increase of 2.2% and 3.1%, respectively. The largest increase in cases overall is seen in the age group of 75–84, with an average of 5% per year (AAPC (95% CI) = 5 (2.3–7.7); *P* = 0.001) (table 1). There was no single point in time where there was a statistically significant reversal in the movement of both mortality rates and the number of deaths from OC (figures 1 and 2).

## Discussion

This study examines the mortality trend caused by OC in Montenegro between 1990 and 2018. On average, 12 individuals die each year in Montenegro from this type of cancer, with a higher number of deaths among males, accounting for 78.5% of cases. The rates have remained stable over the period, but there has been a statistically significant increase in the number of cases for men. The age-standardized rate for the observed period was roughly four times lower than the global average, at 1.34 deaths per 100 000 in the Montenegrin population. This places Montenegro among the countries with the lowest rates, such as the countries of Central America (ASR 0.92), West Africa (ASR 1.2), North Africa (ASR 1.5) and Western Asia (ASR 1.5).^5^

Globally, there has been a decrease in mortality rates from OC over recent decades,^4^^,^^5^^,^^27^ except for the western sub-Saharan region of Africa, where the mortality rate has increased by about 30%. A global analysis of mortality rates over the past 30 years for the same type of cancer revealed no significant differences from 1990 to 1998, followed by a significant increase from 1998 to 2004, a rapid decline from 2004 to 2017, and a gradual increase from 2017 to 2019.^4^

OC is the primary cause of death from cancer in Bangladesh and Malawi. The frequency of OC varies greatly between the two most common histological subtypes. More than half of global OC deaths occur in East Asia, with an average death rate of 12.96 in 2019. Southern and Eastern Sub-Saharan Africa also have higher mortality rates with 11.30 and 10.77, respectively. Malawi has the highest death rate from OC at 25.76. China has the highest number of deaths from OC worldwide, with a reported 257 316 deaths and a mortality rate twice the global average (13.15) in 2019. Mortality rates vary based on the Human Development Index (HDI). Developed countries with lower HDI have higher mortality rates (8.19), while those with medium HDI have slightly lower rates (9.15). The highly developed countries have the lowest mortality rates (3.85).

For men, 21 regions recorded a decline in mortality, while 24 regions had stable trends. The USA, Chile, China, Korea, Colombia and France recorded the most significant decline in mortality rates. On the other hand, three regions had an increasing trend, including Thailand, Latvia and Portugal.^5^

For women, eight regions had a decline in mortality, while 36 regions had stable trends. China, Colombia, USA and Chile recorded the most significant reductions in mortality rates. Only one region, Austria, showed an increasing trend.^5^

During the observed period, Montenegro experienced an OC mortality rate ranging from 0.41 in 1990 to a maximum of 2.34 in 2014, with an average of 1.34 from 1990 to 2018. The last observed year showed a mortality rate of 0.90. In 2019, Nigeria, the Philippines, Peru and Greece recorded the lowest death rates at 1.0, 1.36, 1.38 and 1.39, respectively. Other countries like Egypt, Turkey, Italy and Mexico also had similar rates. For comparison, Serbia had a rate of 2.35^4^ in the same year, with an average age-standardized rate of 1.8 from 1991 to 2015.^28^

The rate of men and women in Montenegro was 2.32/100 000 and 0.51/100 000, respectively, similar to the Belgrade population where they were 2.2 and 0.7. Men in Montenegro had a slightly lower mortality rate of 3.1 compared with the entire population of Serbia. The age groups most affected by death from OC were 55–64 with 30.6% of cases and 65–74 with 31.4% of cases, which is consistent with findings worldwide and also in Serbia. In Serbia, OC mortality rates increased with age, except for women aged 70–79 years. The mortality trend was significant only in men in the 50–59 age group (APC= +1.5%, 95%CI= 0.8–2.3).^28^

In Montenegro, there has been a significant increase in the number of OC cases among males aged 50–64 and 65–74, with an average annual increase of 2.2% and 3.1%, respectively. Additionally, the largest increase in cases was observed in the age group of 75–84, with an average of 5% per year.^28^

In most countries/regions, there has been a decline in mortality from OC in recent years, especially among men and people over 50 years of age. However, in Montenegro, mortality is increasing for the overall population, men, and the elderly population, which is similar to the trend observed in Serbia.^28^

The male-to-female ratio of OC mortality rates in Montenegro is 4.5 on average, while the same ratio for Serbia is 5.2, with higher age-specific mortality rates observed in men in all age groups. Similarly, the rates of OC are generally higher in men than in women across the world.^5^

Neighboring countries like Croatia, Italy, Bulgaria and Slovenia have recorded a significant decrease in OC mortality rates among men, while the trend among women is stable.^5^ In Serbia, the mortality trend for OC has significantly increased among men but not among women.^28^

The overall decrease in mortality is partly due to advancements in diagnostic technology and treatment, such as the detection and treatment of early stage malignant oesophageal tumours by upper endoscopy, neoadjuvant chemotherapy and minimally invasive oesophagectomy.^6^

There are several possible reasons for the reduction in OC mortality rate. These might include a decrease in major risk factors, socioeconomic development, and improved treatment methods. The global prevalence of tobacco and alcohol use has been decreasing since the 1980s, which is believed to have contributed to the overall decline in SCC since the 1990s.^29–31^ It appears that the number of smokers in Montenegro has decreased based on available data.^31^

It is important to strengthen primary prevention measures against risk factors to prevent the development of cancer.^4^ Many of these risk factors are modifiable, providing an opportunity for public health interventions. Preventive measures and clinical treatment vary depending on the histological subtype. Obesity and metabolic syndrome are significant risk factors for EAC, and it is crucial to closely monitor and slow their rising rates. Monitoring the prevalence of obesity and metabolic syndrome is essential, as they pose significant risks for EAC. In Montenegro, there is a growing concern regarding the issue of obesity in children and adults in recent times. Therefore, the Ministry of Health of Montenegro developed a Program of measures to improve the nutritional status and nutrition in Montenegro with the Action Plan from 2021 to 2022, which was created as a continuation of the Program for the Prevention and Control of Chronic Non-Communicable Diseases 2019–2021.^32^

It is already stated that GERB can lead to BE and EAC. To control reflux, proton pump inhibitors (PPIs) are usually prescribed. PPIs have an acid-suppressive effect, as well as potential antioxidant and anti-inflammatory effects, which can help prevent cancer. In Montenegro, PPIs are used according to current recommendations.^19^^,^^20^

To diagnose dysplasia and monitor BE, we follow the ESGE guidelines from 2023.^19^ If a patient is diagnosed with BE with low-grade dysplasia (LGD) on random 4-quadrant biopsies, we repeat the endoscopy. If there are no visible lesions and no advanced pathology [high-grade dysplasia (HGD) or EAC], we suggest follow-up for 6 months.

If LGD is found on repeated endoscopy after 6 months, we use endoscopic eradication therapy (EET). Dysplasia must be confirmed by another experienced pathologist. The risk of progression to HGD/EAC for patients with LGD is between 9.2% and 13.4% per patient per year.^33^^,^^34^

For patients with HGD without a visible lesion, we use ablative techniques to prevent progression to invasive cancer. Radiofrequency ablation (RFA) is the preferred ablative technique among patients undergoing EET. Alternative methods of EET include argon plasma coagulation (APC), hybrid APC and cryoablation. In Montenegro, we have the option of using APC.

EET has revolutionized the management of patients with BE-related neoplasia and offers an effective, minimally invasive treatment approach, avoiding the morbidity and mortality associated with oesophagectomy.^35^ Contemporary practice includes endoscopic resection (ER) of any visible lesion within the BE segment, followed by ablative techniques such as RFA and cryotherapy to achieve complete eradication of dysplasia and reduce the risk of recurrent dysplasia/EAC. In Montenegro, we do not perform any of the mentioned ER techniques yet, but we hope to start soon to prevent unnecessary surgery in the majority of patients.^36^

High-risk patients require complete staging, which may involve endoscopic ultrasound in combination with computerized tomography (CT) or positron emission tomography, and consideration of additional treatments such as chemotherapy and/or radiotherapy and/or surgery.^19^^,^^36^

Montenegro does not conduct screening for BE or EAC, and it is not recommended according to the ESGE guidelines from 2023 due to the low risk in the general population.^19^ The annual risk of progression to HGD or EAC in BE is 0.3–0.8%. Therefore, the cost-effectiveness of the BE screening program is questionable. However, screening can be considered in individuals aged 50 and over with chronic GERD symptoms and in those with at least one risk factor, such as white ethnicity, male gender, obesity, smoking or having a first-degree relative with BE or EAC.^23^^,^^37^^,^^38^

New screening modalities that do not require sedation may be more cost-effective than screening with standard sedated endoscopy.^19^^,^^20^ With the implementation of such modalities, the indications for BE screening/case finding may be expanded in the future.^19^

For the diagnosis of BE in Montenegro, ESGE recommendations are followed. High-definition endoscopes with virtual chromoendoscopy are used before and in addition to Seattle protocol biopsy sampling.^36^

BE ‘indeterminate’ for dysplasia, of any length (confirmed by another pathologist), is advised to repeat oesophagogastroduodenoscopy within 6 months of increasing PPI dosing.^1^

Follow-up of a patient with BE is relative to BE length and dysplasia, as these are both accepted risk factors for disease progression.^19^

Currently, several multifactorial risk estimation tools are being studied to determine the optimal surveillance interval per individual patient, but such tools cannot yet be implemented.^19^

## Conclusion

Monitoring the epidemiologic trend of OC is crucial because it can help formulate more effective public health and clinical strategies, as well as provide etiologic clues. Assessment of recent trends could benefit planning and resource allocation. As technology advances, it is expected that population-based targeted screening endoscopy with more advanced and less invasive technologies will be recommended for high-risk individuals. Montenegro has witnessed a recent increase in the number of deaths, although the mortality rates remain stable. This malignancy appears to impact predominantly two age groups, individuals aged 55–64 and 65–74, which highlights the need for specific attention to be given to this population during the ongoing monitoring of this cancer.

## Data Availability

All data generated or analyzed during this study are included in this article. Further enquiries can be directed to the corresponding author. Key points To our knowledge, this is the inaugural research conducted in Montenegro, which scrutinizes the mortality patterns related to oesophageal cancer (OC). Furthermore, this is the first attempt to investigate measures taken in Montenegro to prevent mortality from this type of cancer. Even though in most countries/regions there has been a decline in mortality from OC in recent years, the mortality rates in Montenegro have remained steady during the investigated period. Unfortunately, a rise in the number of deaths from OC was documented recently in Montenegro, and it seems that OC affects mostly people aged 55–64 and 65–74. Therefore, it is imperative to analyze the trends in mortality associated with OC to create effective prevention strategies.
